# A probabilistic and context-dependent cell culture modelling framework for regulatory toxicology

**DOI:** 10.3389/ftox.2026.1765753

**Published:** 2026-03-24

**Authors:** Eneko Madorran

**Affiliations:** Institute of Clinical and Translational Research, Faculty of Medicine, University of Maribor, Maribor, Slovenia

**Keywords:** context dependency, new approach methodologies (NAMs), organ-based *in vitro* model, probabilistic modeling, translational research, translational toxicology

## Abstract

The persistent gap between preclinical findings and clinical outcomes highlights the limitations of current *in vitro* models in regulatory toxicology. While New Approach Methodologies (NAMs) promise mechanistic insight, reduced reliance on animal testing, and enhanced human relevance, their translational accuracy remains constrained by oversimplified assumptions. This manuscript identifies foundational aspects of human physiology—degeneracy, interconnected pathways, variability among individuals, biological rhythms, and context dependency—that are often underrepresented in existing systems. I propose reframing biological effects as probabilistic outcomes rather than deterministic events, recognizing that cells and organisms operate through overlapping networks where redundancy, variability, and timing shape the likelihood of specific responses. Building on this framework, I outline a probabilistic and context-dependent cell culture model that integrates viability, functional fidelity, pathway mapping, temporal resolution, and Bayesian inference. Translational relevance is further strengthened by anchoring *in vitro* measurements to clinically meaningful benchmarks, incorporating patient perspectives, and aligning with regulatory oversight. Although challenges remain—including replicating cell–cell communication, multi-organ synchronization, and harmonizing probabilistic outputs with deterministic regulatory frameworks—embedding these principles into NAMs offers a pathway to overcome the translational bottleneck. By embracing complexity rather than reducing it, NAMs can evolve into tools that are not only mechanistically informative but also predictive, acceptable, and implementable in real-world milieu, ultimately advancing safer and more ethical toxicological assessment.

## 1 Introduction

The transition toward New Approach Methodologies (NAMs) in regulatory toxicology reflects a growing demand for mechanistic, predictive, and ethically responsible tools that reduce reliance on animal testing while enhancing human relevance (Ouedraogo et al., 2025). Advances in cell-based systems, including organoids and microphysiological platforms, have been widely explored as promising alternatives (Biju et al., 2023; Kim et al., 2020; Lutolf et al., 2024). These approaches aim to capture complex biological processes *in vitro*, offering new opportunities for hazard assessment and mechanistic insight.

Despite their promise, current *in vitro* models often fall short in translational accuracy. Many systems rely on deterministic interpretations of cellular responses, overlooking the inherent variability, redundancy, and context-dependence of human biology (Chunduri and Maddi, 2023; Madorran et al., 2019). This simplification limits the predictive power of NAMs and contributes to the persistent gap between preclinical findings and clinical outcomes (Madorran et al., 2019; Ouedraogo et al., 2025).

The consequences of these limitations are evident in recent clinical studies. The vast majority of successful preclinical studies did not achieve expected outcomes in patients (Polson and Fuji, 2012; Sun et al., 2022). For example, the combination of sodium phenylbutyrate and taurursodiol demonstrated encouraging preclinical efficacy but failed to reproduce results in a clinical amyotrophic lateral sclerosis (ALS) setting (Calcagno et al., 2025). Even the highly promising field of senolytics, which showed striking benefits in preclinical studies (Hickson et al., 2019), yielded divergent results in clinical trials. In humans, Dasatinib plus Quercetin reduced senescent cells in individuals with diabetic kidney disease, yet the effects were far less pronounced than in animal models (Muthamil et al., 2024)—underscoring the importance of real-world data in evaluating translational relevance. These cases illustrate the translational bottleneck: while failures enrich our understanding of disease mechanisms, they also highlight the urgent need to improve experimental models to enhance predictive validity.

At the core of this challenge is the insufficient representation of foundational aspects of human physiology in current *in vitro* systems. This omission penalizes the translation of outcomes to real-world milieu. Although recent efforts to reproduce *in vivo* physiology in cell-based models show promise (Madorran et al., 2024), they still overlook several critical dimensions of human biology. The aim of this work is to identify and discuss these foundational aspects, which remain underrepresented in existing models, and to propose how their integration could improve the translational relevance of NAMs. I hypothesize that embedding a degeneracy, variability, and temporal framework into a hierarchical probabilistic model will increase the translational relevance of NAM-derived *in vitro* data compared with deterministic single-time-point approaches.

## 2 Foundational themes for the new approach methodologies

### 2.1 Biological system

#### 2.1.1 Degeneracy

The term degeneracy in cell system was introduced by Edelman and Gally in 2001, and was described as the ability of elements that are structurally different to perform the same function or yield the same output (Edelman and Gally, 2001). The most straightforward example of cellular degeneracy is DNA translation, where 64 different codon combinations encode just 23 amino acids (Maleszka et al., 2014). This redundancy allows the cell to adapt to diverse conditions, minimize translation errors through codon interchangeability, and overcome limitations in amino acid availability (Maleszka et al., 2014). Another clear example is the immune system, where distinct epitopes derived from a single antigen can activate diverse CD8^+^ T cells that ultimately converge on recognizing the same pathogen (Tieri et al., 2010).

**FIGURE 1:**
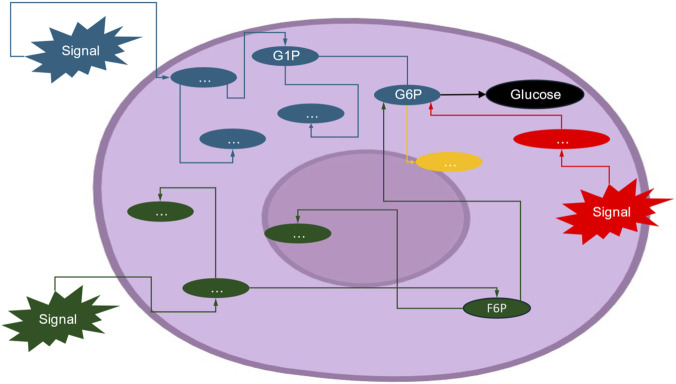
Degeneracy in glucose metabolism. Multiple signals and pathways converge to maintain intracellular glucose availability. Entry routes include glycogen breakdown (blue signal), where glucose-1-phosphate (G1P) is converted into glucose-6-phosphate (G6P), and the pentose phosphate pathway (green signal), where 6-phosphogluconolactone (6-PGL) is also converted into G6P. The red signal represents an additional alternative pathway. The ellipsis (“…”) represents subsequent downstream processes through which these metabolites can be further transformed. Probabilities associated with each branch represent posterior estimates. Thus, this figure illustrates degeneracy by showing how structurally distinct pathways converge on the same metabolic intermediate (G6P), demonstrating that cells can maintain glucose availability through multiple alternative routes. This redundancy underpins robustness and context-dependent flexibility in cellular metabolism. Ultimately, these assumptions define how probability mass propagates through the network and determine whether the test compound’s effects operate independently or through pathway-specific modulation.

Cells also rely on multiple structures and pathways that can yield the same outcome, ensuring metabolic systems remain functional under stress (Edelman and Gally, 2001). Distinct molecular routes can converge on producing the same protein or physiological effect. Cells can generate ATP through glycolysis or oxidative phosphorylation. Each route provides energy, but the sequence chosen determines efficiency and by-products (e.g., lactate vs. CO_2_) (Alberts et al., 2022). A particularly illustrative case is glucose metabolism, where several entry points can feed into the same central pathway. Glucose can be mobilized from glycogen stores via glucose-1-phosphate (G1P), converted into glucose-6-phosphate (G6P), or processed through intermediates such as 6-phosphogluconolactone (6-PGL) in the pentose phosphate pathway (Figure 1). Despite originating from different molecular routes, these intermediates converge to maintain cellular glucose availability and energy balance (Alberts et al., 2022). When glucose availability is limited, cells adapt by switching to alternative fuels. Energy can be derived from fatty acid β-oxidation or from ketone bodies, both of which converge on the production of acetyl-CoA. The choice of pathway has systemic consequences—for instance, during prolonged fasting the brain shifts its fuel preference from glucose to ketone bodies (Boron and Boulpaep, 2012).

Epigenomic regulation provides a clear illustration of degeneracy in biological systems (Whitacre and Bender, 2010). Multiple, structurally distinct mechanisms—such as DNA methylation, histone modifications, chromatin remodelling, and non-coding RNAs—can converge on similar functional outcomes, for example, silencing or activating a gene (Maleszka et al., 2014; Whitacre and Bender, 2010). This overlap ensures robustness, as disruption of one pathway can be compensated by another, yet it also allows divergence under different environmental or developmental conditions. A single transcription factor may regulate different sets of genes depending on chromatin accessibility, co-factor interactions, or tissue-specific circumstances (Maleszka et al., 2014; Whitacre and Bender, 2010). For instance, transcription factors like p53 or NF-κB can orchestrate distinct gene networks in stress response, immunity, or cell survival, demonstrating how one regulator can yield multiple outcomes. At the epigenomic level, degeneracy thus underpins both stability and adaptability, enabling cells to maintain essential functions while retaining the capacity for context-dependent plasticity.

Degeneracy therefore means that cells can reach the same endpoint through multiple routes, but the sequence of processes determines efficiency, regulation, and sometimes even the biological meaning of the outcome (Edelman and Gally, 2001; Maleszka et al., 2014; Yang et al., 2003).

#### 2.1.2 Interconnected pathways - homeostasis

As discussed in the previous chapter on degeneracy, different structural routes can yield similar outcomes (Edelman and Gally, 2001). Thus, various cell pathways share intermediate molecules that link their functions. Yet, cells are also sustained by a dense web of interconnected pathways that not necessarily have similar output. These pathways may have shared intermediate molecules, even originate from the same substrate, but have different outcomes. For example, specific ratios of potassium (K) and calcium (Ca) trigger one response, while altered proportions produce a different outcome (Alberts et al., 2022). Moreover, the end product of one pathway may serve as the substrate for another. This interdependence highlights that cellular processes are not isolated events but components of a dynamic network (Robinson et al., 2020).

**FIGURE 2:**
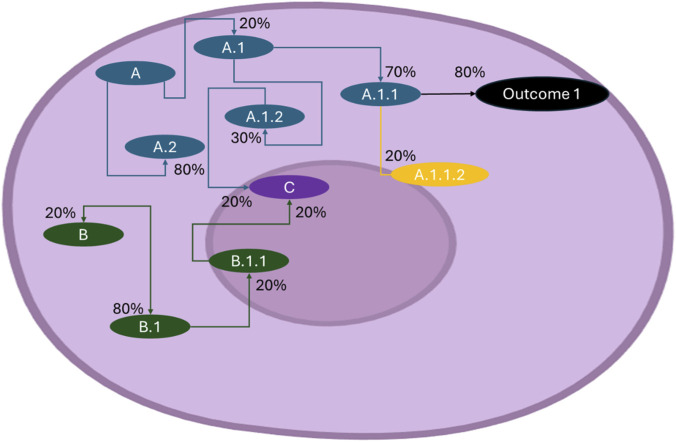
Inference tree of cellular processes. Within the cell, numerous pathways operate simultaneously and are interconnected with one another (A, B and C in the picture). The probability of these processes occurring depends on multiple factors, each of which can alter the likelihood of a given reaction. For example, the probability of reaching outcome 1 through pathway A is obtained by multiplying the conditional probabilities along that branch (P [A.1 ∩ A1.1 ∩ Outcome1] = 11.2%). Factors such as the concentration of solutes or enzymes can shift reactions toward different directions, as illustrated in the transition between B and B.1. This demonstrates that cellular behaviour emerges from chains of probabilistic events rather than single deterministic steps. The probabilities shown are illustrative example values generated from assumed inputs, represent posterior estimates and are included solely to demonstrate how conditional probabilities propagate through the inference tree; they do not represent experimental or model-derived outputs. The example assumes conditional dependence within each pathway branch and conditional independence between branches unless explicitly connected, reflecting how biological processes may converge or diverge.

The overarching purpose of this interconnectedness is the maintenance of homeostasis, regulated thorough receptors, feedback loops, and signalling cascades, which many times entangle opposite pathways, such as glycolysis and gluconeogenesis (Boron and Boulpaep, 2012; Yang et al., 2003). Glycolysis and gluconeogenesis represent opposing metabolic pathways—one breaking down glucose to generate energy, the other synthesizing glucose to maintain systemic supply (Figure 2)—yet they are not simply switched on or off but finely balanced through reciprocal regulation (Boron and Boulpaep, 2012). To prevent futile cycling, key enzymes such as phosphofructokinase-1 (PFK-1) and fructose-1,6-bisphosphatase (FBPase-1) are controlled in opposite directions by allosteric regulators like fructose-2,6-bisphosphate and the cellular energy charge (ATP/AMP ratios). High AMP levels drive glycolysis when energy is scarce, whereas elevated ATP and citrate shift metabolism toward gluconeogenesis when energy is abundant (Metallo and Vander Heiden, 2013). Hormonal signals further tune this balance: insulin promotes glycolysis, while glucagon favours gluconeogenesis (Boron and Boulpaep, 2012). Importantly, both pathways can be partially active at the same time, with their relative “signal strengths” determining the net metabolic outcome. This conditional overlap allows the liver and other tissues to adapt flexibly—providing intermediates for biosynthesis, maintaining blood glucose, and ensuring metabolic robustness without wasting energy (Alberts et al., 2022). In essence, the coexistence of glycolysis and gluconeogenesis at different intensities exemplifies how interconnected pathways achieve precision through feedback, amplification, and context-dependent regulation.

Beyond intracellular regulation, the extracellular matrix (ECM) plays a critical role in sustaining homeostasis. The ECM is not merely a structural scaffold but an active regulator of cell behaviour, influencing adhesion, migration, differentiation, and survival (Hynes, 2009; Theocharis et al., 2016). ECM components such as collagen, laminin, and fibronectin interact with integrins and other receptors to transmit mechanical and biochemical signals into the cell, a process known as mechanotransduction. These signals regulate cytoskeletal organization, gene expression, and metabolic activity, thereby linking tissue architecture to cellular physiology (Frantz et al., 2010). Importantly, ECM remodelling is dynamic: matrix metalloproteinases (MMPs) degrade ECM components, while fibroblasts and other cells synthesize new matrix proteins, which allows tissues to adapt to stress, injury, or development. Yet, excessive ECM remodelling can disrupt homeostatic boundaries and lead to pathological states such as fibrosis or cancer (Lu et al., 2011). Thus, the ECM functions as a central hub for maintaining tissue homeostasis, integrating mechanical stability with biochemical regulation.

Finally, while homeostasis is often discussed at the level of single organs, it is inherently systemic and depends on cell–cell communication (CCC) across tissues (Su et al., 2024). For example, clotting factors synthesized in the liver act within the vascular system, where platelets from the bone marrow and endothelial cells contribute essential cofactors and regulatory signals (Alberts et al., 2022). The coagulation cascade exemplifies this interconnectedness: thrombin not only converts fibrinogen to fibrin but also feeds back to activate upstream factors, while anticoagulants such as antithrombin III and protein C restrain excessive clotting. This balance ensures stability—strong enough to prevent haemorrhage, yet controlled to avoid thrombosis (Su et al., 2024). CCC thus represents the systemic dimension of homeostasis, where signals and substrates circulate between organs to sustain physiological equilibrium.

#### 2.1.3 Variability among individuals

Just as cells achieve robustness through degeneracy and interconnected pathways, organisms exhibit variability in how physiological systems function across individuals. This variability arises from a combination of genetic polymorphisms, epigenetic regulation, and environmental influences, which together shape differences in physiology and drug response (Collins et al., 2003).

Genetic polymorphisms provide a foundational explanation for interpersonal differences. As highlighted by Collins et al. (2003), variation in alleles encoding enzymes, receptors, and transporters can alter physiological processes such as metabolism, hormone signalling, and immune function. These polymorphisms explain why individuals with otherwise similar physiology may differ in baseline traits such as blood pressure, glucose tolerance, or neurotransmitter activity (Collins et al., 2003).

Epigenetic regulation and environment add further complexity. Lifestyle factors such as diet, stress, and exposure to toxins can modify gene expression through DNA methylation, histone modifications, or non-coding RNAs (Alegría-Torres et al., 2011). These changes may amplify or attenuate the effects of genetic polymorphisms, meaning that two individuals with the same genotype can still exhibit distinct physiological responses depending on their environmental history (Evans and McLeod, 2003).

Systems-level interactions integrate molecular layers into metabolic outcomes. As Civelek and Lusis (2014) highlight, gene–environment interactions are not confined to transcriptional or proteomic changes but extend deeply into metabolic networks, where fluxes of metabolites reflect the dynamic state of cellular physiology. Individual variability therefore arises less from isolated genetic variants than from the coordinated regulation of metabolic pathways, shaped by inherited architecture and environmental inputs such as diet, activity, and exposure (Civelek and Lusis, 2014). This metabolic integration underscores how networks of transcripts, proteins, and metabolites collectively determine phenotypic diversity and disease susceptibility (Civelek and Lusis, 2014).

Drug response variability is a direct consequence of these differences. Evans and McLeod (2003) demonstrated that polymorphisms in drug-metabolizing enzymes (e.g., cytochrome P450 isoforms), drug transporters, and receptor proteins can profoundly influence both pharmacokinetics and pharmacodynamics (Evans and McLeod, 2003). As a result, a compound that is therapeutic in one individual may be ineffective or even toxic in another. This principle underpins the field of pharmacogenomics and the drive toward personalized medicine (Sadee et al., 2023).

In summary, variability among individuals reflects the convergence of genetic diversity, epigenetic modulation, and environmental situation (Civelek and Lusis, 2014; Collins et al., 2003; Evans and McLeod, 2003). Recognizing this variability is essential for interpreting physiological data, understanding disease progression, and tailoring therapeutic interventions. Just as degeneracy and interconnected pathways confer robustness at the cellular level, interpersonal variability highlights the adaptability and individuality of human physiology.

Noteworthy, it is important to note that differences between animal and human models exceed the variability observed among individuals. These differences extend beyond physiology to cellular composition and structure (Madorran et al., 2019). For example, human pancreatic islets contain 30%–50% alpha cells dispersed throughout, whereas rodent islets contain only 10%–20% alpha cells, typically restricted to the periphery (Campbell and Newgard, 2021).

#### 2.1.4 Biological rhythm

We must also consider that biological systems are not static; they operate within rhythmic cycles that profoundly influence physiology. As Roenneberg and Merrow (2016) emphasize, circadian clocks orchestrate cellular and systemic processes, ensuring that gene expression, protein synthesis, and metabolism are aligned with the day–night cycle (Roenneberg and Merrow, 2016). This temporal structuring means that the same genetic program can yield different outcomes depending on when it is activated, underscoring time as a critical determinant of biological function.

Gene expression fluctuates across the day, with circadian rhythms driving distinct transcriptional and protein synthesis profiles during daytime versus nighttime phases (Roenneberg and Merrow, 2016). Certain genes are expressed only at specific times, and proteins involved in metabolism, repair, or signalling follow rhythmic patterns of synthesis and degradation (Ayyar and Sukumaran, 2021). This temporal compartmentalization ensures efficiency and prevents conflicting processes from occurring simultaneously. Importantly, the sequence of gene activation matters: the order in which one gene is upregulated before another can fundamentally alter downstream pathways. Even subtle shifts in timing can reshape physiological responses, who show that circadian timing influences pharmacological and therapeutic outcomes (Ayyar and Sukumaran, 2021).

Circadian regulation extends beyond transcription and translation to encompass metabolic pathways. Core clock genes such as BMAL1 and CLOCK coordinate glucose and lipid metabolism, ensuring that energy balance aligns with feeding–fasting cycles (Poggiogalle et al., 2018). Disruption of these rhythms can lead to metabolic disorders, including obesity and diabetes. Drug metabolism itself is circadian-modulated: enzymes responsible for detoxification and clearance fluctuate in activity across the day, altering pharmacokinetics. Thus, both endogenous metabolism and exogenous drug processing are tightly bound to biological time (Poggiogalle et al., 2018).

The temporal dimension raises critical questions for experimental and therapeutic contexts. Studying the effect of a drug on a cell *in vitro* may not capture its full impact *in vivo*, where circadian cycles and systemic cues modulate responses. The textbook Chronobiology: Biological Timekeeping (2004) stresses that circadian clocks synchronize across tissues, meaning that drug absorption, distribution, metabolism, and elimination vary depending on the time of administration (Chronobiology: Biological timekeeping, 2004). This field of chronopharmacology demonstrates that the efficacy and toxicity of drugs are not fixed properties, but dynamic outcomes shaped by biological rhythms. For example, primary human hepatocytes had different inflammatory response to LPS, IFN-β, or Plasmodium infection when exposing them at different circadian times (March et al., 2024). Furthermore, many studies show how drugs often have significantly different pharmacokinetics and pharmacodynamics when administered at specific circadian phases (Butler et al., 2024; Gutu et al., 2025; Hermida et al., 2007).

Importantly, physiology also evolves with aging, which interacts with circadian regulation and metabolic homeostasis. Aging is associated with dampened circadian amplitude, altered sleep–wake cycles, and reduced responsiveness of clock genes, leading to impaired synchronization of metabolic pathways (Hood and Amir, 2017; Farhud and Aryan, 2018). These changes contribute to age-related declines in glucose tolerance, lipid handling, and immune function (Bulterijs et al., 2015; Haigis and Sinclair, 2010; Kane and Sinclair, 2019). Yet, degeneracy and homeostatic mechanisms often maintain apparent health until thresholds are exceeded (Kitano, 2004; Alberts et al., 2022). This raises a practical question: do age-related physiological changes always translate into perceived illness, or can compensatory mechanisms sustain function despite underlying pathway alterations? Evidence suggests that many older individuals remain asymptomatic until stressors—such as infection, toxin exposure, or metabolic overload—reveal the diminished resilience of aged systems (Lei et al., 2022; López-Otín et al., 2013).

Thus, both circadian rhythms and aging trajectories must be considered when interpreting physiological variability. Biological processes are not only environment-dependent but also time-dependent, shaped by daily cycles and long-term changes across the lifespan. Integrating chronobiology with aging research provides a more complete framework for understanding health, disease progression, and therapeutic intervention.

### 2.2 Context dependency

#### 2.2.1 Effect of the environment

Cellular responses are not determined solely by genetic information; they are profoundly shaped by the surrounding environment. Even subtle variations in ion concentrations can alter how a cell responds to the same signal or input, demonstrating that context can decisively influence physiological outcomes (Alberts et al., 2022). This principle extends beyond the cellular level to whole organisms, where environmental conditions interact with genetic architecture to shape behaviour and physiology.

A landmark study by Crabbe et al. (1999) highlighted this phenomenon in mice (Crabbe et al., 1999). Despite tightly controlled laboratory conditions, genetically identical animals displayed significant differences in physiological and behavioural outcomes across different sites. Importantly, the strength of genetic effects varied between laboratories, showing that genotype–environment interactions actively reshape outcomes rather than simply introduce random noise. For example, the distance travelled by mice differed markedly between facilities, underscoring how subtle environmental factors can alter experimental results. These findings emphasize that outcomes may reflect not only genetic determinants but also the interplay between genotype and laboratory-specific conditions. In some cases, environmental influences may even outweigh the tested substance itself.

The implications of environmental modulation extend further into gene regulation and therapeutic response. Environmental factors can alter gene expression, as observed in serotonin regulation during sensitive developmental windows (Booij et al., 2015). This means that therapeutic interventions may vary in effectiveness depending on the circumstance in which they are applied. Critical questions therefore arise: to what extent do environmental conditions alter disease progression, and how might they influence the success or failure of treatments?

Systems genetics approaches suggest that such complexity arises from networks of transcripts, proteins, and metabolites that integrate genetic and environmental inputs to produce context-dependent outcomes (Civelek and Lusis, 2014). Epigenetic modifications add another layer of regulation, influencing transcription through mechanisms such as DNA methylation, histone modifications, and non-coding RNAs, that may even be translated later in life (Poggi et al., 2026; Whitacre and Bender, 2010). Importantly, degeneracy in regulatory mechanisms provides both robustness—ensuring stability under perturbation—and evolvability—enabling innovation and adaptation over time (Whitacre and Bender, 2010).

In summary, environmental context is not a secondary factor but a central determinant of physiological and therapeutic outcomes. Recognizing the interplay between genetic, epigenetic, and environmental influences is essential for interpreting experimental data, understanding disease progression, and designing interventions. Just as degeneracy and interconnected pathways confer robustness at the cellular level, environmental modulation highlights the adaptability and individuality of biological systems, framing the broader theme of how stability and flexibility are sustained in living organisms.

#### 2.2.2 Effect of time

Observing a system at only one moment can misrepresent its true trajectory. For example, measuring blood glucose immediately after a meal may suggest hyperglycaemia, yet without follow-up monitoring it would miss the subsequent insulin-mediated decline and risk misclassifying the patient’s metabolic status. Similarly, a drug that appears safe at an early time point may later reveal toxicity once compensatory mechanisms are exhausted. Physiological responses to drugs or toxic compounds are inherently time-dependent, and their impact varies according to the stage of homeostatic balance in which they occur.

Clinically, timing shapes therapeutic outcomes. In oncology, tumour profiling before treatment may capture a driver mutation that is later lost through clonal selection, highlighting the need for longitudinal monitoring (Al Bakir et al., 2025; Greaves and Maley, 2012; McGranahan and Swanton, 2017). Likewise, the physiological state of an organ changes with age, circadian rhythms, diet, or inflammation, meaning that the same intervention may have different effects depending on when it is applied.

Metabolic processes further illustrate this principle. Under perturbation, cells may shift between glycolysis, oxidative phosphorylation, lipid oxidation, or redox buffering. These transitions reveal whether pathways adapt, compensate, or collapse over time (Liu et al., 2025; Mardinoglu and Nielsen, 2015; Palsson, 2015). Toxicity is thus not static but evolves as metabolic networks respond to stress, underscoring the need for longitudinal metabolic mapping.

This temporal dependency extends to the molecular scale. Sequential gene expression, activation of signalling cascades, and metabolite fluxes can rewire pathway outputs, meaning that the timing of signals relative to one another determines the eventual physiological outcome (Kitano, 2004; Nielsen, 2017). Mapping signal-to-output timing is therefore essential to uncover hidden mechanistic steps and to understand pathway complexity. Without such resolution, single time-point observations risk faulty conclusions about the effect of a drug or toxin.

Categorizing toxic effects by temporal windows—immediate, delayed, cumulative, or recovery—provides a framework for distinguishing reversible insults from progressive damage. Acute exposures may appear harmless initially but manifest harm once compensatory mechanisms fail, while severe insults can be reversible if repair and regeneration outpace injury. Duration is equally important: short-lived actions may be advantageous when synchronized with circadian peaks of target activity, whereas sustained effects may be required to bridge low-activity phases (Ayyar and Sukumaran, 2021). These distinctions emphasize that both the timing and persistence of compound effects determine therapeutic efficacy.

This raises a fundamental question: what defines toxicity in relation to time? Should harm be classified only when permanent lesions occur, or should transient insults also be considered toxic if they disrupt cellular physiology, even temporarily? Conversely, how should exposures be interpreted when no immediate effect is observed, but long-term damage emerges later? Addressing these questions requires integrating temporal dynamics into both experimental design and clinical monitoring. By combining systems biology approaches with longitudinal profiling, researchers can better capture the evolving nature of toxicity and therapeutic response, ensuring that interventions are evaluated not only by their immediate impact but also by their long-term consequences.

### 2.3 Probability

A central question in systems biology is whether cellular responses operate in a probabilistic fashion, where the likelihood of a given outcome increases with the presence of required subsets of molecules (Friedman, 2004). Cells can be conceptualized as networks of probabilistic events. Each activation step (A, A.1, A1.1 or A, A.1, A.1.2, …) contributes to an inference tree that reveals how pathways are connected (Friedman, 2004; Robinson et al., 2020). Constructing such probabilistic response trees allows researchers to estimate the likelihood of specific outcomes and to identify hidden dependencies between pathways (Friedman, 2004). Implementing these dependencies requires empirical data, and recent work on tipping points within adverse outcome pathways provides a conceptual and quantitative foundation for identifying the specific outcomes (Simon and Becker, 2025). Integrating tipping-point concepts also enables the incorporation of organism-level feedback control loops, which are often overlooked (Simon and Becker, 2025). Integration of prior knowledge with observed data to refine predictions of pathway connectivity and output probabilities may be achieved by Bayesian regression (Friedman, 2004; Yugi et al., 2016). For example, Bayesian modelling of metabolic networks has shown that fluxes are better represented as probability distributions rather than fixed values (St John et al., 2019; Tran et al., 2008) (Figure 2).

Another layer of complexity arises when cells receive simultaneous inputs. Probabilistic frameworks of metabolic associations show how simultaneous inputs reshape outcome distributions, emphasizing the need for robust sampling (Rosato et al., 2018). The number of possible outcomes expands combinatorially, and the relative probabilities shift depending on the nature and timing of these inputs (Rosato et al., 2018). For instance, if a system allows for ten possible outcomes but only five models are available, predictions will inevitably lack precision. This highlights the importance of scaling experimental models: with too few cells or insufficient replicates, the full distribution of outcomes cannot be captured, and the resulting model will misrepresent proportions (Madorran et al., 2019).

**FIGURE 3:**
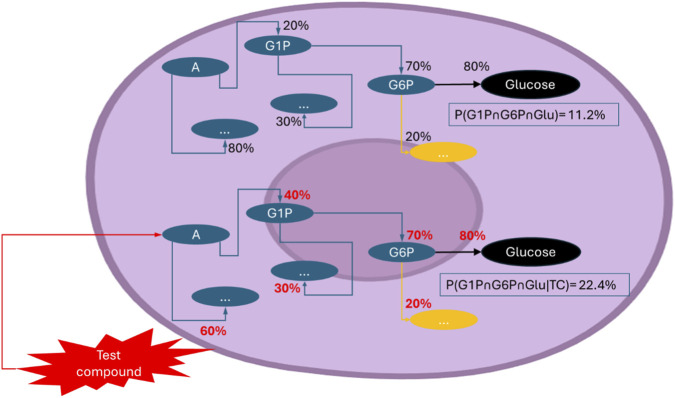
Probability shifts induced by the test compound. The diagram illustrates how a test compound modifies the likelihood of specific cellular events, revealing whether its effects act independently or rely on existing pathways to shape the biological response. For clarity, the test compound and glucose are abbreviated as TC and Glu in the probability expressions. In this case, the test compound increases the probability of glucose-1-phosphate (G1P) formation within the A pathway, thereby influencing the probability of downstream glucose production. Each percentage represents the probability of transitioning from one intermediate to the next (e.g., A → G1P → G6P → glucose). The overall probability of glucose formation is obtained by multiplying the conditional probabilities along the branch (P [G1P∩ G6P ∩ Glu]. In this example, the test compound increases the probability of G1P formation (P [G1P∩ G6P ∩ Glu|TC]), which doubles the overall probability of downstream glucose production (from 11.2% to 22.4%). Crucially, this effect did not result from changes in ATP availability—an essential requirement for glucose synthesis—indicating that it occurred independently. The probabilities shown are illustrative example values generated from assumed inputs and are included solely to demonstrate how conditional probabilities propagate through the pathway; they do not represent experimental or model-derived outputs.

Bayesian inference thus emerges as a unifying framework for embedding probabilistic cellular behaviour into biological models (Friedman et al., 2000). By integrating prior expectations with experimental observations, it enables researchers to estimate outcome likelihoods under diverse conditions and to refine these estimates as new data accumulate (Liu et al., 2025; Yugi et al., 2016). In physiological contexts, this approach reframes cellular and organ-level outputs as probability distributions rather than fixed templates, capturing the inherent variability of biological systems (Babtie and Stumpf, 2017). Importantly, probabilistic modelling highlights how stochastic fluctuations and incomplete pathway activation can dampen maximal responses, underscoring the need for uncertainty-aware frameworks in systems biology (Babtie and Stumpf, 2017). Bayesian parameter inference methods for stochastic biochemical networks (Golightly and Wilkinson, 2011) further provide tools to quantify uncertainty and iteratively improve predictions. Recent advances in probabilistic modelling of biochemical reaction systems have expanded these capabilities, enabling more efficient inference and more realistic representation of stochastic cellular dynamics (Le Coënt et al., 2024). As datasets expand, posterior probabilities converge toward more accurate representations of biological reality, revealing whether the tested compound acts independently or in dependence on existing pathways to shape the expected biological response (Golightly and Wilkinson, 2011; Le Coënt et al., 2024) (Figure 3). This probabilistic framing is particularly relevant for drug discovery, as compounds with genuine efficacy may be discarded too early if their effects are masked by stochastic variability in cellular responses (Golightly and Wilkinson, 2011).

## 3 Probabilistic and context-dependent cell culture model

We previously examined key features of human physiology alongside well-established mathematical principles that govern their behaviour. These concepts are widely cited and recognized as foundational to understanding the human organism. However, current *in vitro* models often overlook these assumptions, despite their critical importance for translating laboratory findings into real-world applications. The objective, therefore, is to develop a cell-based model that not only yields consistent *in vitro* outcomes across diverse culture conditions but also incorporates these essential physiological and mathematical insights. By aligning the model more closely with *in vivo* physiology this approach enhances the translational relevance of experimental data. Ultimately, the proposed framework bridges the gap between cell culture systems and real-world experience, enabling more robust and predictive toxicological assessments.

### 3.1 Replicating *in vivo* environments

A central challenge in experimental biology is whether the complex environment observed *in vivo* can truly be recreated *in vitro*, and for how long such effects can be sustained. Cellular responses *in vitro* frequently diverge from *in vivo* outcomes because culture systems impose constraints that do not exist physiologically, including limited nutrient availability (e.g., calcium or micronutrient fluctuations), altered cell density, disrupted biological rhythms, and reduced biological variability when relying on a single cell line. Moreover, *in vitro* systems are typically highly controlled and homogeneous, capturing only a narrow slice of the variability present in real-world biological environments. Testing across multiple settings is therefore essential to distinguish direct biological effects from artifacts of the experimental environment. To improve translational relevance, several key characteristics must be considered when designing cell models:

- Cell numbers and density: Models must contain sufficient cell populations to represent organ physiology. Adequate cell mass is also required for downstream analyses such as proteomics (Kassem et al., 2021). Microfluidic systems or organoids with very small populations may fail to yield measurable proteomic data, limiting their utility.
- Coculture systems: Incorporating multiple cell types allows more realistic intercellular communication. However, coculture inherently adds complexity to the model, and shifts in coculture dynamics or homeostatic balance can alter cellular responses and influence how experimental outcomes are interpreted.
- Extracellular matrix (ECM): The ECM must be comparable to that observed *in vivo*. Ideally, fibroblasts or stromal cells within the target organ should produce the ECM, rather than relying solely on external supplementation, to ensure physiological relevance. As discussed above, the dynamic interactions between cells and their surrounding ECM are fundamental for maintaining physiological function and should be faithfully incorporated into *in vitro* models using fibroblasts.
- Cell culture medium: Although many experiments aim to recreate identical environments by maintaining a constant culture medium, cells *in vivo* are continuously exposed to dynamic and fluctuating conditions. Because of degeneracy, slight variations in the environment may still lead to comparable functional outcomes. Evaluating cellular responses across different media therefore becomes essential, as it reveals whether a sample performs consistently under varied conditions. Incorporating this variability may be key to more faithfully reproducing the complexity of the *in vivo* environment.
- Model scale and event coverage: The physical dimensions and overall scale of the culture system increase the probability of observing the full spectrum of reactions. Larger substrates and higher total cell numbers enable spatial gradients, rare events, and collective dynamics that cannot be captured in small-scale systems, aligning *in vitro* dynamics more closely with *in vivo* physiology.
- Physiological benchmarks: To ensure translational accuracy, *in vitro* models must be anchored to clinically measurable parameters of human origin. Examples include insulin secretion in pancreatic cultures or albumin production in liver systems (Madorran, 2025). These markers provide a direct link between experimental outcomes and human physiology, allowing models to reflect both healthy and diseased states. Clinical experts - mainly pathologists - play a critical role in this alignment by evaluating cell morphology, differentiation status, and overall model organization, since cells that lose their physiological identity no longer serve as reliable proxies for *in vivo* tissue.

Building on this foundation, systems genetics approaches should be incorporated to capture the complexity of genotype–environment interactions. Networks of transcripts, proteins, and metabolites reveal how cellular responses are shaped by both inherited architecture and external influences. At the same time, population variability must be considered: differences in tissue size, organ function, and systemic resilience across individuals can significantly alter outcomes, and ignoring this diversity risks overgeneralization. Analytical frameworks that incorporate redundancy and noisy-channel model help manage this biological variability, ensuring that observed outcomes reflect genuine physiological processes rather than experimental noise.

Under these circumstances, results must be interpreted in relation to tissue function rather than cell number alone, since functional capacity is the true determinant of physiological relevance.

Finally, adopting a streamlined analytical flow—clinical diagnostics define the benchmarks, *in vitro* systems are engineered to meet them, *in vitro* analyses reveal mechanistic insights, and these insights can then be applied back to clinical perspective to refine understanding of human physiology and disease:

- Establishing healthy physiology *in vitro* is the first step. Baseline ranges should be defined by identifying markers of wellbeing in healthy samples, similar to routine clinical checks in patients. These baselines then serve as reference points against which dysfunction can be measured.
- From there, pathophysiology can be modelled by inducing disease states *in vitro*. Dysfunctional markers—such as altered enzyme activity, receptor failure, or impaired signalling—should be compared directly with clinical data to validate the model’s relevance. This approach ensures that *in vitro* systems not only mimic normal physiology but also reproduce pathological conditions in a way that is clinically meaningful.
- Finally, toxicity testing must move beyond the narrow definition of cell death. A cell that survives but fails to perform its physiological duty is equally compromised. Conversely, an initial toxic insult may be temporarily compensated or repaired by intrinsic cellular mechanisms. At the tissue level, organisms may further buffer dysfunction—for example through hyperplasia or increased metabolic load—masking early damage and creating a misleading impression of preserved health, or, in some cases, restoring function well enough to fully recover the homeostasis balance. Toxicity assessment should therefore include markers of altered physiology—enzymes, proteins, lipids—and evaluate whether cells behave as their *in vivo* counterparts. Importantly, communication between different cell types (e.g., hepatocytes and endothelial cells) must be incorporated to reflect organ-level interactions, since toxicity often emerges from systemic rather than isolated cellular responses.

### 3.2 Measuring the model

Accurate measurement is the cornerstone of translational relevance. To bridge experimental observations with clinical outcomes, *in vitro* models must capture not only whether cells survive, but whether they continue to perform their physiological function. This requires a framework that integrates viability, function, pathway mapping, probabilistic modelling, and temporal resolution.

#### 3.2.1 What to measure

Viability, function and context dependency: Distinguish between cells that remain alive but lose function versus those that maintain homeostasis. A cell may survive exposure to a toxic agent yet fail to fulfil its *in vivo* functions, such as hormone secretion or metabolic regulation. Moreover, a minimum set of contextual variables must accompany all measurements to ensure that viability and functional readouts are interpretable within the modelling framework.

- Cell viability: Viability remains a fundamental measure, particularly in toxicology, since cell death following exposure to a compound represents the most straightforward indicator of toxicity.
- Homeostasis: Beyond mere survival, it is essential to determine whether cells continue to perform their physiological functions as they would *in vivo*. While cells may recover following stress, restored viability does not guarantee full functional integrity, as downstream consequences can still emerge over time. Monitoring renewal capacity and sustained function is therefore critical to capture resilience and long-term stability. In some cases, compensatory mechanisms—such as hyperplasia—may maintain performance without leading to lasting detrimental effects, underscoring the need to evaluate both immediate recovery and long-term adaptation.
- Minimum context set: To ensure that viability and functional readouts are interpretable, a minimal set of contextual variables must be recorded. This includes cell source or donor, culture medium composition, extracellular matrix or substrate, oxygenation conditions, and the exposure calendar, with flow or co-culture parameters added when relevant. These variables represent the smallest set of environmental determinants known to influence cellular behaviour. Capturing them ensures that observed effects—whether loss of function, recovery, or sustained homeostasis—reflect underlying biology rather than uncontrolled shifts in experimental setting.

In addition to viability, homeostasis and minimum context set, the model should incorporate broader dimensions:

- Clinical anchoring: Relate measurements to real-world data, especially clinical trials, to ensure that *in vitro* findings reflect patient outcomes.
- Pathway engagement: Determine how many pathways a compound influence. Using pathway atlases, trace how a molecule from the extracellular environment enters the cell, is processed, and may exit unchanged or incorporated into larger complexes. This mapping reveals whether effects are confined or systemic.
- Concentration: The concentration of compounds should be expressed as the number of molecules per cell. As emphasized by Madorran et al. (2019), it is the intracellular molecules that ultimately drive the cellular response (Madorran et al., 2019).

#### 3.2.2 How to measure

All outcomes in cellular models can be expressed probabilistically. Each readout—viability, pathway activation, functional loss, or recovery—corresponds to a probability *p* of observing a given state under defined conditions. Bayesian inference provides a structured way to update these probabilities when moving from baseline (control) conditions to perturbed (test) conditions, while explicitly quantifying uncertainty.

- Data: In this framework, D denotes the observed data, consisting of viability measurements, functional fidelity markers, and pathway-activation readouts collected across donors, batches, laboratories, and time points. The data D is the full dataset encompassing = {D_control_, D_test_}.
- Priors: Prior probabilities π(Hi) represent the expected likelihood of each hypothesis before observing new data. They are informed by baseline measurements collected across multiple control conditions—different media, donor origins, exposure timings, and other sources of biological and environmental variability. This broader sampling refines the priors, reduces the risk of experimental setting specific artefacts, and increases confidence that observed effects reflect genuine biology. When no prior evidence exists, uninformative priors such as π(H_0_) = 0.5 (compound not toxic) and π(H_1_) = 0.5 (compound toxic) may be used; when relevant studies exist, priors should instead be weighted according to their similarity to the current system.
- Likelihood: The likelihood P (D|H_i_) represents the probability of observing the experimental data D under each hypothesis H. In our setting, H has two values: H_1_ - the test compound is toxic, H_0_ – the test compound is not toxic. In this framework, the data consist of viability and pathway activation measurements, modelled hierarchically to incorporate donor, batch, laboratory, and temporal effects. This structure ensures that both technical and biological variability are represented in the probability function.
- Posterior: Posterior probabilities P(Hi|D) answer the central decision question: “Given the observed data, how likely is it that the compound produces an adverse effect?” Uncertainty is expressed through the posterior distribution and can be summarised using credible probability bands that map directly onto the decision framework introduced later in the manuscript.
- Control vs. test probabilities: Control conditions define the baseline probability of an outcome under normal functioning. Test conditions represent the conditional probability of the same outcome given a perturbation. A meaningful change in probability indicates an effect; unchanged probabilities suggest independence between the perturbation and the outcome.
- Bayes’ theorem:
$$P\;\left(H_{i}\left|D\right.\right)=\frac{P\;\left(\left.D\right|H_{i}\right)\cdot \;\pi \;\left(H_{i}\right)}{P\left(D\right)}$$
  where:
$$P\;\left(D\right)=P\;\left(D\left|\;H_{0})\;\cdot \pi \left(H_{0}\right)+P\;(D\right|H_{1}\right)\;\cdot \pi \left(H_{1}\right)$$
- Dimensional scaling: Determine the minimum dimensions of the cell model at which outcomes remain proportional across all factors. In this sense, the number of cells within the model is crucial, and should be big enough to represent all the potential outcomes, so the outcomes on this model will be representative on the real-world data. This ensures probabilities calculated per cell are representative and scalable.
- Probabilistic interpretation: RNA expression levels can exhibit substantial increases (e.g., ten-fold) without necessarily producing biological effects. A probabilistic framework helps ensure that such changes are interpreted in a context set rather than presumed to be functionally significant.

#### 3.2.3 When to measure

Timing is as crucial as the measurement itself, since a single snapshot may misrepresent the actual state—as discussed in the Section 2.2.2, glucose levels taken immediately after a meal can give a misleading picture.

- Time-resolved analysis: Many toxicological processes are missed without temporal mapping. Capturing the duration of processes leading to cell death or recovery provides mechanistic clarity:
T outcome−T beginning=∑processes
- Temporal sustainment: Drug responses, signalling cascades, or sustained interventions (e.g., laser stimulation) may persist differently depending on physiological state. Without accounting for time, models risk misrepresenting durability or trajectory.
- Minimum temporal design. To make timing operational rather than conceptual, the modelling framework requires a minimal temporal design. Single time points risk misrepresenting dynamic processes, so at least two post-exposure measurements are needed to capture directionality, with additional sampling aligned to known biological phases when rhythmic behaviour is expected. Repeated measurements enter the hierarchical Bayesian model as time-indexed observations, allowing the framework to distinguish transient from sustained effects and to quantify temporal trajectories. In each point, the minimum context set should be measured.

##### 3.2.3.1 Working example

This simple worked example illustrates how interacting probability distributions may operate within the proposed Bayesian framework. In this example, the minimum-viable workflow is made explicit so that each modelling step corresponds to a concrete laboratory action.

- Required inputs
  - Minimum context set: Cell source/donor, medium composition, ECM/substrate, oxygenation, exposure calendar, flow or co-culture parameters when applicable.
  - Functional readouts: Viability, functional fidelity (organ-specific markers), pathway engagement, concentration expressed as molecules per cell.
  - Temporal schema: At least two post-exposure time points to capture directionality, with additional phase-aligned sampling when circadian or biological rhythms are expected.

ii. Model family
  - A hierarchical Bayesian structure with donor, batch, laboratory, and time as random effects; compound exposure as a fixed effect.
  - Likelihood functions defined for each readout; priors informed by multi-context control data.

iii. Outputs
  - Posterior probability of an adverse effect.
  - 95% credible interval.
  - Calibration target (posterior predictive check pass rate).

iv. Decision mapping
  - High concern (P > 0.80): classify as adverse or proceed to higher-tier testing.
  - Intermediate (0.40 < P < 0.80): request orthogonal evidence or extended temporal sampling.
  - Low concern (P < 0.40): no concern; waive further testing.

This worked example demonstrates how Bayesian components interact to generate interpretable, decision-relevant outputs and prepares the ground for the structured decision-mapping framework described in the following section, under Section 3.4.

### 3.3 Real world environment

The ultimate goal of preclinical research is not only to generate mechanistic insight but to ensure that findings can be translated into meaningful outcomes for real-world settings. Achieving this requires a framework that integrates what to measure, how to measure, and when to measure, while also embedding clinical expertise, patient perspectives, and regulatory oversight.

#### 3.3.1 From viability to function

Translation begins with recognizing that cell survival alone is insufficient. Cells must maintain homeostasis and perform their physiological functions—whether hormone secretion, metabolic regulation, or structural support. Clinical experts such as pathologists or internists play a crucial role in evaluating whether cells and tissues behave as they would *in vivo*. For example, in a pathophysiological model, the organ should resemble its diseased state, including compensatory mechanisms such as hyperplasia. This ensures that models reflect not only life or death but also functional fidelity.

#### 3.3.2 Patient integration and stakeholder perspectives

Enhancing translational relevance requires the systematic incorporation of patient-derived information. Patients are not passive recipients but active stakeholders whose experiences and expectations shape the feasibility of future interventions. Contemporary patient-engagement research highlights that involving patients early in the translational pipeline improves the relevance of research questions, strengthens endpoint selection, and reduces the risk of developing interventions that are scientifically promising but clinically impractical (Carroll et al., 2022). Patient input helps clarify organism-level effects of pathologies induced by compounds or disease, complementing mechanistic insights from preclinical models. Equally important, understanding desired outcomes from the patient perspective is critical. For instance, if a therapy requires repeated biopsies every 6 months, willingness to participate may be limited. Likewise, if a treatment causes side effects such as hair loss in 80% of patients, recruitment into clinical studies may be unfeasible regardless of preclinical promise. Effective translation necessitates a careful equilibrium between mechanistic efficacy, patient acceptability, and applicability within real-world clinical setting, consistent with emerging frameworks that position patient engagement as a core component of high-quality translational research.

**FIGURE 4:**
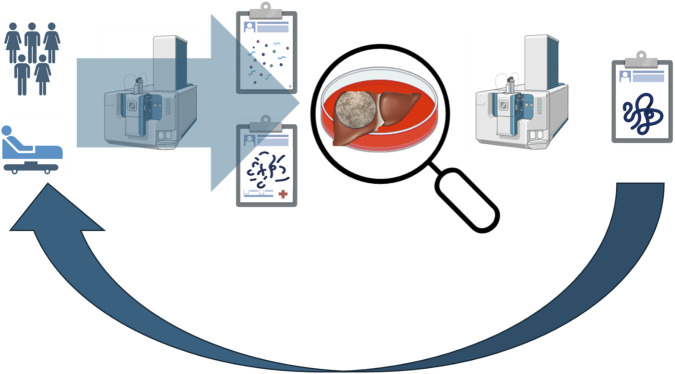
New Approach methodology workflow. Physiological data from populations and patients—such as albumin synthesis, triglyceride–glucose dynamics, and hepatic drug biotransformation for a liver model—are collected to build an organ model that closely mirrors *in vivo* function. Within the *in vitro* system, markers of organ (patho)physiology can be identified with reduced background noise. These biomarkers are subsequently translated into real-world applications. Clinical diagnostics define the benchmarks, *in vitro* systems are engineered to meet them, *in vitro* analyses reveal mechanistic insights, and these insights can then be applied back to clinical perspective to refine understanding of human physiology and disease.

A concrete example of structured patient engagement comes from Norway, where St. Olavs Hospital has implemented systematic patient involvement in treatment planning and decision-making. In addition, their emerging precision-oncology approach—using each patient’s own cancer cells to identify the most suitable therapy—illustrates a real-world application that aligns with the workflow proposed in Figure 4 (Sliper Midling, 2025).

#### 3.3.3 Validation

Before any regulatory consideration, peer validation should be conducted through coordinated inter-laboratory studies that repeat the same experimental conditions across different sites, donors, and platforms. Ideally, these experiments may also be conducted in the EU Reference Laboratory for alternatives to animal testing (EURL ECVAM). These datasets enable explicit modelling of batch, donor, laboratory, and temporal effects, allowing the framework to quantify and separate technical variance from true biological variability. External benchmark sets—derived from well-characterized compounds, reference materials, or published datasets—provide an additional layer of evaluation and help assess the model’s generalizability beyond the original training setting. Validation also requires demonstrating that posterior probabilities remain stable when experimental conditions vary within realistic bounds and that the hierarchical structure correctly captures shared patterns while preventing overfitting.

Once a method enters regulatory use, validation becomes an ongoing process: laboratories using the method should openly share their experience, reproducibility metrics, encountered issues, and optimizations, supported by real-world data and continuous peer exchange.

This two-stage approach—pre-regulatory peer validation followed by post-regulatory community validation—ensures that the model remains robust, transparent, and adaptable as new evidence accumulates.

To strengthen generalizability beyond a qualitative description, the validation logic incorporates explicit quantitative criteria, including posterior predictive check pass rates, probability calibration curves comparing predicted and observed frequencies, and performance on external benchmark compounds evaluated under distributional shift. These metrics provide measurable indicators of model robustness and support transparent validation across laboratories and circumstances.

For toxicological models, engagement with competent authorities is essential. Regulators ultimately decide whether a method can be implemented, and their early involvement helps align experimental design with future application, a topic developed further in the following section.

### 3.4 New approach methodologies in regulatory toxicology

The integration of NAMs into regulatory toxicology represents both an opportunity and a challenge. NAMs promise mechanistic insight, reduced reliance on animal testing, and enhanced human relevance, yet their adoption requires alignment with regulatory frameworks that prioritize reproducibility, transparency, and public safety. In this sense, regulatory toxicology serves as the bridge between experimental innovation and societal implementation, ensuring that novel models meet the standards necessary for clinical, environmental protection and public-health protection.

For NAMs to be accepted, rigorous validation is essential. Models must demonstrate reproducibility by yielding consistent results across laboratories, culture conditions, and populations. Outcomes must be benchmarked against clinically relevant parameters to ensure translational accuracy. Standard operating procedures are needed to harmonize protocols, minimize variability, and guarantee comparability across studies. International collaboration further strengthens this process, with organizations such as OECD and ECVAM playing a central role in developing guidelines for validation and acceptance. OECD guidance and EURL ECVAM GIVIMP principles emphasize that new *in vitro* and computational approaches must be developed, reported, and validated using transparent decision criteria, standardized method documentation, rigorous uncertainty report, and reproducible laboratory practices to ensure regulatory fitness for purpose (European Commission. Joint Research Centre, 2023; OECD, 2018; Pamies, 2021). In parallel, the ICCVAM Strategic Roadmap (NTP-ICCVAM-ROADMAP2018) highlights the need for human-relevant science, transparent uncertainty report, performance-based validation, and early regulatory engagement to support the adoption of NAM (Interagency Coordinating Committee on the Validation of Alternative Methods, 2018). Navigating regulatory approval requires NAMs to fit within established pathways. Often, NAM data are integrated into weight-of-evidence approaches, complementing existing toxicological evidence to reinforce risk assessments. In certain circumstances, regulators have accepted NAMs for specific endpoints—for example, skin sensitization under Test Guideline 442C or genotoxicity under the Micronucleus (TG 487) and Comet (TG 489) assays (OECD, 2016; OECD, 2010) —while continuing to require additional evidence to address systemic or chronic effects.

Regulatory use of probabilistic models requires that continuous posterior probabilities be translated into clear, deterministic actions. To support this, probability outputs can be mapped onto predefined decision probability bands. High posterior probabilities of an adverse effect would trigger protective regulatory actions, such as hazard classification or progression to higher-tier testing. Intermediate probability bands would indicate the need for additional evidence—such as orthogonal assays, mechanistic markers, or expanded temporal sampling—before a regulatory conclusion can be reached. Low-probability outcomes would support a “no concern” determination and justify waiving further testing. Uncertainty would be communicated through credible probability bands, posterior predictive checks, and sensitivity analyses, in line with current expectations for transparent uncertainty reporting.

Ultimately, regulatory toxicology provides the framework through which NAMs can transition from experimental innovation to societal application. By embedding validation, clinical anchoring, probabilistic interpretation, and stakeholder engagement, NAMs can achieve regulatory acceptance and deliver on their promise of safer, more ethical, and more predictive toxicological assessment.

### 3.5 Predictions and intended use

1. Probabilistic models that incorporate degeneracy, inter-individual variability, homeostatic feedback, and environmental context will better capture biological variability than deterministic single-time-point approaches.
2. Models that explicitly integrate temporal dynamics—including circadian and biological rhythms—will reduce unexplained variance and improve predictive accuracy relative to single-time-point designs.
3. A hierarchical probabilistic model that separates donor, batch, and laboratory effects will demonstrate greater generalizability across datasets than deterministic models, which typically conflate biological and technical sources of variation.
4. Context-aware probabilistic outputs will reduce false-positive and false-negative classifications in hazard identification and potency ranking compared with context-agnostic approaches.

The intended use for this framework is hazard identification and potency ranking within regulatory toxicology.

### 3.6 Limitations

Despite its potential, the proposed model presents several important limitations that must be acknowledged, both to clarify its current boundaries and to guide future refinements.

One of the most critical challenges lies in replicating the complexity of cell–cell communication. *In vivo*, communication occurs across diverse cell types, enabling coordinated signalling, regulation, and adaptation. Organ-based models, however, often lack the full repertoire of interacting populations, which compromises the fidelity of observed responses.

A second limitation concerns the inability to reproduce the synchronized functioning of multiple organs. Without incorporating temporal synchronization, even mechanistically sound models may fall short in predicting real-world responses, as they fail to capture the systemic rhythms that shape metabolism, signalling, and therapeutic outcomes.

Data interpretation also remains a challenge. Probabilistic outcomes and context-dependent variability complicate regulatory decision-making, which has traditionally relied on deterministic thresholds. This mismatch between probabilistic biological behaviour and deterministic regulatory frameworks can hinder acceptance and practical application.

Finally, global harmonization poses a significant barrier. Divergent regulatory standards across regions slow adoption and limit the utility of NAMs in international environment, creating inconsistencies in how models are validated, applied, and recognized.

Addressing these limitations will require the integration of multi-cellular communication networks and the embedding of temporal dynamics into model design. Advances in multi-organ systems and synchronized modelling approaches may help overcome current constraints, ultimately enhancing the translational accuracy of preclinical findings and strengthening the regulatory acceptance of NAMs.

The workflow outlined in Figure 4 represents a conceptual pathway for capturing information from clinical studies and examining it under more controlled conditions using *in vitro* organ-based models. Its purpose is to illustrate how clinically observed patterns can be decomposed, interrogated mechanistically, and ultimately translated back into clinically meaningful insights. This framework can support applications beyond toxicology—including the identification and refinement of clinically relevant biomarkers, therapeutic-response profiling or to understanding biomarker behaviour across temporal and physiological contexts. The minimum-viable workflow presented in this manuscript is actionable and intended for immediate laboratory implementation. Practical deployment of this workflow—including assay selection, data-integration pipelines, and validation procedures——it is not described in full operational detail here, as such implementation requires laboratory-specific SOP development.

## 4 Conclusion

The persistent gap between preclinical findings and clinical outcomes underscores the need for a fundamental change in the mindset of the preclinical environment. Traditional approaches often emphasize tightly controlled conditions and statistical significance as the benchmark for evaluating compounds. While such rigor is valuable for assessing the influence of a test compound on a particular pathway, it can be detrimental to real-world translation. Clinical and environmental contexts rarely replicate the controlled conditions of preclinical models, and the multitude of variables influencing outcomes *in vivo* cannot be ignored.

This manuscript has highlighted several foundational aspects of human physiology—degeneracy, interconnected pathways, variability among individuals, biological rhythms, and context dependency—that are often underrepresented in current *in vitro* systems. These dimensions are not peripheral but central to understanding how biological systems function and adapt. Their omission penalizes translational accuracy, contributing to the poor predictability of preclinical models.

A key proposal advanced here is to reframe biological effects as probabilistic outcomes rather than deterministic events. Cells and organisms operate through networks of overlapping pathways, where variability, redundancy, and timing shape the likelihood of specific responses. By embedding probabilistic modelling into experimental design, researchers can better capture the inherent ambiguity and diversity of biological systems. This perspective allows outcomes to be interpreted not as fixed endpoints but as distributions shaped by biological, environmental, and temporal factors.

Furthermore, translation requires anchoring *in vitro* measurements to clinically meaningful benchmarks, integrating patient perspectives, and aligning with regulatory oversight. Viability alone is insufficient; models must assess whether cells maintain homeostasis and perform their physiological functions. Pathway mapping, temporal resolution, and probabilistic interpretation provide the tools to bridge experimental data with real-world outcomes.

In sum, this work proposes that probabilistic modelling, grounded in foundational physiological principles, is essential for improving translational accuracy in toxicology. Thus, the future of preclinical research lies in embracing complexity rather than reducing it. By incorporating foundational physiological principles, probabilistic frameworks, and stakeholder perspectives, NAMs and other advanced models can evolve into tools that are not only mechanistically informative but also predictive, acceptable, and implementable in real-world milieu. This shift in mindset—from deterministic precision under artificial conditions to probabilistic accuracy under realistic variability—offers a pathway to overcome the translational bottleneck and to ensure that experimental findings contribute meaningfully to human health and safety.

## Data Availability

The original contributions presented in the study are included in the article/supplementary material, further inquiries can be directed to the corresponding author.
